# Associating Appendicitis with Metabolic Dysfunction-Associated Steatotic Liver Disease (MASLD): A Novel Insight into an Unexpected Connection

**DOI:** 10.3390/jcm13051319

**Published:** 2024-02-26

**Authors:** Christoph Roderburg, Dirk Waldschmidt, Catherine Leyh, Sarah Krieg, Andreas Krieg, Tom Luedde, Sven H. Loosen, Karel Kostev

**Affiliations:** 1 Department of Gastroenterology, Hepatology and Infectious Diseases, University Hospital Düsseldorf, Medical Faculty of Heinrich Heine University Düsseldorf, Moorenstrasse 5, 40225 Düsseldorf, Germany; catherine.leyh@med.uni-duesseldorf.de (C.L.); sarah.krieg@med.uni-duesseldorf.de (S.K.); sven.loosen@med.uni-duesseldorf.de (S.H.L.); 2 Department of Gastroenterology, University Hospital of Cologne, 50937 Cologne, Germany; 3 Department of General and Visceral Surgery, Thoracic Surgery and Proctology, University Hospital Herford, Medical Campus OWL, Ruhr University Bochum, 44801 Bochum, Germany; andreas.krieg@klinikum-herford.de; 4 Epidemiology, IQVIA, 60549 Frankfurt, Germany

**Keywords:** appendicitis, NAFLD, MASLD, MASH, metALD, epidemiology

## Abstract

Background: The gut microbiome modulates the liver immune microenvironment and is deeply integrated into the pathophysiology of metabolic dysfunction-associated steatotic liver disease (MASLD). Appendectomies, which are performed in almost all patients diagnosed with appendicitis, cause long-term alterations to the gut microbiome, providing a potential link with the development of MASLD. We therefore investigated a potential link between appendicitis and the presence of MASLD in a large cohort of outpatients in Germany. Methods: The present study included 26,717 individuals with and 26,717 without appendicitis. Univariable Cox-regression analyses were conducted to assess the association between appendicitis and MASLD. Results: During the long-term follow-up, 4.8% of patients with appendicitis and 3.4% of those in the non-appendicitis group were diagnosed with MASLD (*p* < 0.001), corresponding to an incidence of 5.4 (appendicitis cohort) versus 3.5 (non-appendicitis cohort) cases per 1000 patient years. These findings were confirmed in regression analysis, revealing a strong and statistically significant association between appendicitis and the development of MASLD (HR: 1.57; 95% CI: 1.39–1.78). This link was observed for all age groups and was independent of patients’ sex. Conclusion: We provide evidence from a large cohort of outpatients in Germany suggesting a link between appendicitis and MASLD. This might help to better stratify patients according to their individual risk for the development of chronic liver diseases.

## 1. Introduction

In recent years, as the prevalence of MASLD has risen to alarming levels, researchers have sought to understand the many factors that contribute to the disease. Among other factors, the so-called “gut–liver axis” plays a critical role in the development and progression of MASLD. Compelling evidence has directly linked alterations in the gut microbiota to the development and disease severity of MASLD [1,2,3,4].

Appendicitis, a common surgical emergency affecting millions of people worldwide, has historically been viewed as an isolated condition, with its resolution by appendectomy typically considered a definitive cure [5,6]. Recently, several biochemical markers including IL-6 (which is synthesized in the liver) and bilirubin have been established to predict a complicated clinical course, e.g., due to organ perforation [7,8,9]. A growing body of research has shed new light on the long-term consequences of appendicitis beyond the acute phase. The appendix is a reservoir for a large number of microorganisms in the human body [10]. The appendix harbors abundant biofilms, consistently releasing bacteria into the intestinal lumen. Its microbiota rivals the diversity found in the colon and actively influences the gut microbiota. Multiple studies propose a potential link between appendicitis and the onset of inflammatory conditions like inflammatory bowel disease (IBD), heart disease, and even unexpected conditions such as Parkinson’s disease [11,12,13]. An appendectomy was further identified as a negative risk factor for recurrent Clostridium difficile infection, as an important nosocomial infection in hospitals [14]. Based on these findings, we hypothesized that appendicitis and subsequent appendectomy may be a risk factor for the development of MASLD. By investigating the complex relationship between appendicitis and MASLD, we hope to contribute to a more nuanced understanding of the pathogenesis of MASLD and provide novel approaches to risk assessment, prevention and management in patients after appendectomy due to appendicitis.

## 2. Materials and Methods

### 2.1. Database

This retrospective cohort study was based on data from the IQVIATM Disease Analyzer database, which contains anonymous electronic medical records from computer systems used in the practices of general practitioners and specialists in Germany. The database contains data on demographics, treatments and diagnoses from approximately 3000 office-based physicians in Germany. The panel of practices included in the Disease Analyzer database has previously been shown to be representative of office-based physicians in Germany. In recent years, this data source has been used in studies focusing on different epidemiological topics, including MASLD [15,16,17].

### 2.2. Study Population

**Figure 1:**
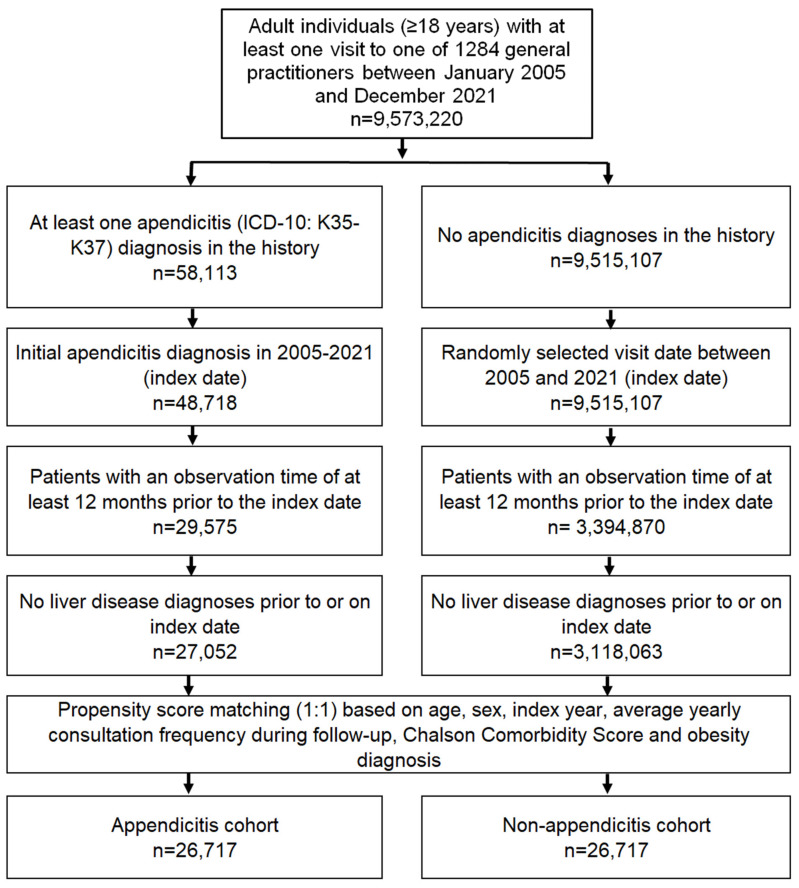
Selection of study patients.

This study included adult individuals with a first diagnosis of appendicitis (ICD-10: K35–K37) in 1284 general practices in Germany between 01/2005 and 12/2021 (index date; Figure 1).

All patients had an observation period of at least twelve months before the index date. Patients with a diagnosis of liver disease (ICD-10: B18, K70-K77) before inclusion were excluded to estimate the association between appendicitis and MASLD without the influence of other liver diseases. After applying similar inclusion criteria, individuals without appendicitis were matched to those with appendicitis using nearest neighbor propensity score matching (1:1) based on age, sex, index year, average annual consultation frequency during follow-up, baseline diagnosis of obesity as a major risk factor for MASLD, and Charlson comorbidity score [18]. For the non-appendicitis cohort, the index date was that of a randomly selected visit between January 2005 and December 2021 (Figure 1). The inclusion of average annual consultation frequency during follow-up was needed to avoid selection bias, as patients with appendicitis may visit physicians more frequently after appendicitis therapy.

### 2.3. Study Outcomes and Statistical Analyses

The outcome of the study was the initial diagnosis of NAFLD/MASLD (ICD-10: K75.8, K76.0) in the up to 10 years after the index date as a function of appendicitis. Differences in the sample characteristics and diagnosis prevalence between the appendicitis and non-appendicitis cohorts were compared using the Wilcoxon signed-rank test for continuous variables and the McNemar and the Stuart–Maxwell tests for categorical variables. The 10-year cumulative incidence of MASLD in the appendicitis and non-appendicitis cohorts was analyzed using Kaplan–Meier curves and compared using the log-rank test. Univariable Cox regression analyses were performed to assess the association between appendicitis and MASLD in the total cohort, in the five age groups, in women and men, as well as in patients with and without diabetes and obesity, separately. A *p*-value of <0.05 was considered statistically significant. Analyses were performed using SAS version 9.4 (SAS Institute, Cary, NC, USA).

## 3. Results

### 3.1. Basic Characteristics of the Study Sample

The present study included 26,717 individuals with and 26,717 individuals without appendicitis, identified using the IQVIATM Disease Analyzer database, which contains anonymous electronic medical records from computer systems used in the practices of general practitioners and specialists in Germany. The baseline characteristics of the study patients are shown in Table 1.

In summary, the mean age was 40.5 (SD: 17.9) years in the appendicitis cohort and 40.6 (SD: 17.9) years in the non-appendicitis group. Overall, 56% of patients were female. The prevalence of obesity (BMI > 25 kg/m^2^) was 7.5% in both groups of patients. Patients had an average of 6.0 visits per year during follow-up, with no difference seen between the two cohorts of patients. The mean Charlson Comorbidity Score (CCS) was 1.0 in both groups. Most patients were enrolled between 2017 and 2021 (index years).

### 3.2. Association of Appendicitis with a Subsequent Diagnosis of MASLD

**Figure 2:**
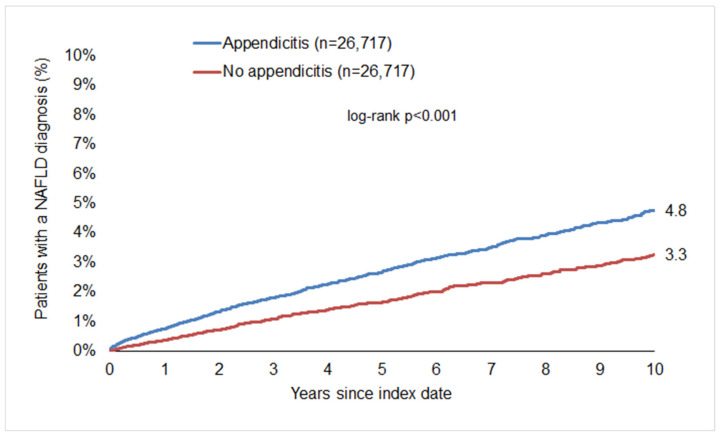
Cumulative incidence of MASLD in patients with and without appendicitis.

After up to 10 years of follow-up, 4.8% of patients in the appendicitis cohort were diagnosed with MASLD, compared to only 3.3% of the non-appendicitis cohort (*p* < 0.001, Figure 2), clearly showing that appendicitis represents a previously unrecognized risk factor for the development of MASLD in humans.

This corresponds to an incidence of 5.4 (appendicitis cohort) versus 3.5 (non-appendicitis cohort) cases per 1000 patient years. Subsequent regression analysis showed that there was a significant association between appendicitis and a subsequent MASLD diagnosis in the total population (HR: 1.57; 95% CI: 1.39–1.78), highlighting the role of appendicitis as a novel risk factor for the development of MASLD.

### 3.3. Age- and Sex-Stratified Analyses

In age-stratified analyses, the association between appendicitis and MASLD was significant in all age groups but was strongest in the 31–40 years age group (HR: 1.75; 95% CI: 1.29–2.38). Recent data suggest a modification of risk factors associated with MASLD by patient sex. Similarly, the association between appendicitis and MASLD was stronger in women (HR: 1.74; 95% CI: 1.46–2.09) than in men (HR: 1.44; 95% CI: 1.22–1.70) but reached the prespecified level of significance in both groups (Table 2).

## 4. Discussion

By analyzing a total of approximately 50,000 patients (26,717 with and 26,717 without appendicitis), our study shows that a history of appendicitis is associated with the development of MASLD. Confirming previous data on gender differences in the pathophysiology of MASLD, the association between appendicitis and MASLD was stronger in women than in men. Notably, these findings represent the first data from a large outpatient cohort in Germany on this association, potentially establishing appendicitis as a novel risk factor for MASLD.

An appendectomy, which is performed in almost all patients diagnosed with appendicitis, is a common surgical procedure, carrying potential risks and complications, both short and long term. Short-term problems, such as infection and intra-abdominal abscess, are generally manageable in routine clinical practice [5,6,10]. However, the long-term complications associated with appendicitis and appendectomy are not well understood. Recently, researchers have shown that patients who have undergone appendectomy may be at increased risk of developing Crohn’s disease, ulcerative colitis, Clostridium difficile infection, sepsis and colorectal cancer [11]. In line with this, Nakano et al. found a significantly higher frequency of appendectomy history in MASLD patients with advanced fibrosis, suggesting a potential risk of MASLD progression to MASH and cirrhosis [19]. However, until now, the role of appendectomy as a trigger for MASLD itself remained unexplored. Taken together with the data from Nakano et al. [19], our data add to the current understanding of the pathophysiology of MASLD. The gut microbiota, a diverse community of microorganisms residing in the gastrointestinal tract, plays a crucial role in maintaining homeostasis and influencing metabolic processes [1,2,3,4]. Interestingly, the effect of appendicitis on the development of later MASLD was most pronounced in the age group of patients between 31 and 40 years. It must be said that no direct comparison can be made between the different age groups, so it remains unclear whether this observation is due to chance or not. One possible explanation is that many patients in this age group have their first contact with screening examinations and are therefore more likely to be diagnosed in this age group. Perturbations in the composition and function of the gut microbiota—such as those observed following appendectomy—may contribute to increased gut permeability, facilitating the translocation of microbial products, such as lipopolysaccharides, to the liver. This in turn triggers inflammatory cascades and metabolic derangements, exacerbating hepatic steatosis and inflammation. In addition, the gut microbiota is actively involved in the metabolism of dietary components and produces bioactive molecules that affect host physiology, including hepatic lipid metabolism [1,2,3,4]. In a recent exploration of the correlation between appendectomy and intestinal immunity, Juan et al. revealed that appendectomy results in a significant reduction in serum sIgA levels. The reduction in sIgA levels following appendectomy diminishes the intestinal defense capacity, potentially leading to an elevation in gut-derived endotoxins due to bacterial overgrowth and compromised intestinal barrier integrity [20]. In summary, understanding the dynamic relationship between the gut microbiota and MASLD provides a promising avenue for therapeutic interventions aimed at modulating the microbiome to mitigate the progression of this prevalent liver disorder.

Our study’s key strength lies in the extensive patient cohort, bolstering the robustness and scientific credibility of our findings. Additionally, the use of a previously validated database known for its representativeness [21], and its application in various studies on inflammatory and malignant diseases [9,10,17,18,19], enhances the reliability of our research. However, it is crucial to recognize notable limitations influencing the interpretation of our study outcomes. The reliance on ICD-10 codes for all diagnoses may lead to misclassification and undercoding of certain conditions. Our study was specifically designed to examine the association between appendicitis and MASLD, adjusting for age and sex as confounders. Unfortunately, certain data elements essential for further analysis were missing, such as socioeconomic status, genetic predisposition, environmental factors, lifestyle choices (e.g., physical activity, tobacco or alcohol consumption, dietary habits), and other variables associated with an increased risk of MASLD. Furthermore, the database used does not contain information on mortality. Finally, there was no information on the hospital treatment of appendicitis, namely surgery. Although consistent with previous research, our study can only establish associations, not causal relationships. Despite these limitations, our study is the first to present data from a large German outpatient cohort treated in German practices between 2004 and 2022.

Considering the large number of people at risk of developing MASLD worldwide, the identification of patients with a particularly high probability of developing the disease itself or developing complications is crucial. Current developments are moving in the direction of so-called metabolic boards in which individual patients are discussed on an interdisciplinary basis—similar to what we know from tumor patients. Our data, which identify appendicitis as a new risk factor, fit in perfectly with this trend.

## 5. Conclusions

In conclusion, we present, for the first time, data from a large German primary care database showing that appendicitis is associated with an increased incidence of MASLD. Thus, our study suggests that previous appendicitis should be recognized as a risk factor for the development of MASLD and for the progression of MASLD to MASH or cirrhosis in order to improve long-term outcomes in these patients. Such data could be helpful in risk prediction and in defining individualized approaches to prevention, for example in “metabolic panels”, as suggested by current expert recommendations. On a mechanistic level, further research is needed to better understand the detailed mechanisms linking appendicitis and MASLD.

## Figures and Tables

**Table 1:** Baseline characteristics of the study sample (prior to and after 1:1 propensity score matching).

	Prior to Matching			After Matching		
Variable	Proportion Among Appendicitis Patients (*n*, %) *n* = 27,052	Proportion Among Non-Appendicitis Patients (*n*, %) *n* = 3,118,063	*p*-Value	Proportion among Appendicitis Patients (*n*, %) *n* = 26,717	Proportion Among Non-Appendicitis Patients (*n*, %) *n* = 26,717	*p*-Value
Age (Mean, SD)	40.6 (17.9)	51.6 (19.5)	<0.001	40.5 (17.9)	40.6 (17.9)	0.855
Age 18–30	10,264 (37.9)	561,734 (18.0)		10,155 (38.0)	10,082 (37.7)	0.926
Age 31–40	4758 (17.6)	452,655 (14.5)		4687 (17.5)	4760 (17.8)	
Age 41–50	4767 (15.4)	484,665 (15.5)	<0.001	4185 (15.4)	4128 (15.5)	
Age 51–60	3640 (13.5)	547,496 (17.6)		3609 (13.5)	3592 (13.4)	
Age > 60	4223 (15.6)	1,071,513 (34.4)		4141 (15.5)	4155 (15.6)	
Female	15,149 (56.0)	1,677,793 (53.8)	<0.001	14,950 (56.0)	14,906 (55.8)	0.702
Male	11,903 (44.0)	1,440,270 (46.2)		11,767 (44.0)	11,811 (44.2)	
Obesity diagnosis	2179 (8.1)	245,839 (7.9)	0.300	1994 (7.5)	1982 (7.4)	0.843
Number of physician visits per year during the follow-up (Mean, SD)	6.0 (3.9)	5.7 (4.3)	<0.001	6.0 (3.9)	6.0 (3.9)	1.000
Charlson Comorbidity Score (CCS) (Mean, SD)	1.1 (1.6)	1.4 (1.9)	<0.001	1.0 (1.5)	1.0 (1.5)	0.439
CCS 0	12,459 (46.1)	1,306,208 (41.9)		12,418 (46.5)	12,522 (46.9)	0.918
CCS 1	8081 (29.9)	811,788 (26.0)		8043 (30.1)	7963 (29.8)	
CCS 2	3173 (11.7)	412,356 (13.1)	<0.001	3128 (11.7)	3129 (11.7)	
CCS 3	1510 (5.6)	235,214 (7.5)		1471 (5.5)	1464 (5.5)	
CCS > 3	1829 (6.8)	352,497 (11.3)		1657 (6.2)	1639 (6.1)	
Index year 2005–2008	2751 (10.2)	282,330 (9.1)		2683 (10.0)	2704 (10.1)	0.127
Index year 2009–2012	4894 (18.1)	391,249 (12.5)		4787 (17.9)	4699 (17.6)	
Index year 2013–2016	7133 (26.4)	661,754 (21.2)	<0.001	7035 (26.3)	6855 (25.7)	
Index year 2017–2021	12,274 (45.3)	1,782,730 (57.2)		12,212 (45.7)	12,459 (46.6)	

Proportions of patients given in *n*, %, unless otherwise indicated. SD: standard deviation.

**Table 2:** Association between appendicitis and subsequent MASLD diagnoses in patients followed in general practices in Germany (univariable Cox regression models).

Patient Group	Incidence (Cases per 1000 Patient Years) in the Appendicitis Cohort	Incidence (Cases per 1000 Patient Years) in the Non-Appendicitis Cohort	HR (95% CI)	*p*-Value
Total	5.4	3.5	1.57 (1.39–1.78)	<0.001
Age 18–30	2.1	1.4	1.42 (1.02–1.96)	0.035
Age 31–40	5.3	3.0	1.75 (1.29–2.38)	<0.001
Age 41–50	7.2	4.7	1.54 (1.20–1.98)	<0.001
Age 51–60	9.4	5.7	1.67 (1.30–2.14)	<0.001
Age ≥ 60	7.4	5.1	1.53 (1.18–1.99)	0.002
Female	4.7	2.7	1.74 (1.46–2.09)	<0.001
Male	6.2	4.4	1.44 (1.22–1.70)	<0.001
Obesity	14.1	9,2	1.58 (1.19–2.10)	0.002
No obesity	4.7	3.0	1.57 (1.37–1.80)	<0.001

## Data Availability

The datasets used and/or analyzed during the current study are available from the corresponding author upon reasonable request.
